# Easily Biodegradable Organic Carbon Release in the Deep Bed of Slow Sand Filters

**DOI:** 10.1021/acsestwater.5c00932

**Published:** 2025-10-15

**Authors:** Shreya Ajith Trikannad, Jan Peter van der Hoek, Yuwei Huang, Doris van Halem

**Affiliations:** † Department of Water Management, 2860 Delft University of Technology, Building 23, Stevinweg 1, 2628 Delft, The Netherlands; ‡ Eawag, Swiss Federal Institute of Aquatic Science and Technology, 8600 Dübendorf, Switzerland; § Waternet, Korte Ouderkerkerdijk 7, 1096 AC Amsterdam, The Netherlands

**Keywords:** drinking water production, biofiltration, biological stability, carbon cycling, nitrification

## Abstract

Slow sand filters (SSFs) are increasingly recognized for enhancing the biological stability of drinking water. While research has historically focused on the top layer (*Schmutzdecke*) of SSFs, the contribution of deeper filter depths in removing dissolved organic carbon (DOC) and ammonium (NH_4_^+^) has recently been acknowledged. This study investigated the occurrence and potential pathways of DOC release in mature full-scale, and young laboratory SSFs. The top layer (5 cm) reduced the easily biodegradable DOC, mainly low-molecular-weight (LMW) acids and building blocks. The middle layers (20–60 cm) released DOC, particularly LMW acids and neutrals, at depths where nitrification was nearly complete. This release occurred in both mature and young SSFs and may result from bacterial activity under carbon or nitrogen limitation or from the transformation of slowly degradable DOC into labile forms. Whatever the precise mechanism of release, the bottom layers (60–90 cm) subsequently removed this released DOC and reduced PO_4_^3–^ to ultralow levels, highlighting the importance of the deepest layers in maintaining effluent quality. This study provides the first evidence of biodegradable DOC release in SSFs and emphasizes the need to better understand its implications for carbon cycling and removal processes in biological filters.

## Graphical abstract

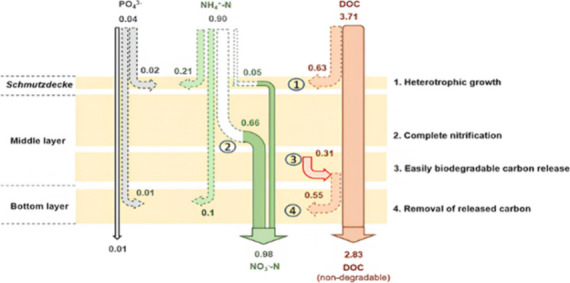

## 1 Introduction

The supply of microbially safe and high-quality drinking water is a fundamental responsibility of every water utility. To achieve this, distributed drinking water should be biologically stable, ensuring that water quality remains unchanged from the treatment plant to the customer’s tap. Water utilities are increasingly concerned about biodegradable fractions of dissolved organic carbon (DOC), ammonium (NH_4_^+^), and phosphate (PO_4_^3–^) in drinking water. These compounds can negatively impact microbiological water quality and encourage the growth of pathogens in the distribution network of unchlorinated systems and act as precursors to disinfection byproducts in chlorinated systems.^1^ To address these concerns, utilities have relied on advanced treatment methods, such as membrane filtration (e.g., reverse osmosis), ozonation, and advanced oxidation processes (AOPs). However, slow sand filtration, traditionally valued for its disinfection capabilities, has gained prominence for its ability to remove DOC and NH_4_^+^ effectively during drinking water treatment.^2^

DOC in water comprises of refractory (i.e., poorly biodegradable), slowly biodegradable, and assimilable organic carbon (AOC) fractions.^3,4^ Biodegradable DOC and AOC in drinking water can stimulate bacterial regrowth during distribution.^5^ In addition, slowly biodegradable DOC, such as biopolymers, negatively affect the biological stability of drinking water.^6^ SSFs remove DOC by a combination of physicochemical and biological processes.^7,8^ Biological removal is driven by diverse microbial communities that develop within biofilms throughout the filter bed.^9,10^ Heterotrophic bacteria utilize both easily and slowly biodegradable DOC for their growth. While NH_4_^+^ removal is driven biologically by nitrifying bacteria that oxidize NH_4_^+^ to nitrite (NO_2_^–^) and nitrate (NO_3_^–^).^11^

Until recently, the key processes and microbial communities responsible for the overall functionality of SSFs were considered to occur primarily within the biologically active top layer, called the *Schmutzdecke.* However, recent studies have challenged this traditional focus on the *Schmutzdecke* by emphasizing the role of deeper filter layers in pathogens and nutrient removal.^2,12,13^ In mature full-scale SSFs, DOC removal occurred primarily in the top layer, and NH_4_^+^ removal occurred in the deeper depths.^2^ Notably, after *Schmutzdecke* removal through scraping, the deeper layers effectively reduced DOC and NH_4_^+^, suggesting that the deeper sand bed supports the long-term stability and resilience of SSFs by compensating for disturbances in the top layers. In line with these findings, another study demonstrated that glucose removal was not confined to the *Schmutzdecke* but distributed throughout the filter column.^13^ Specific bacterial groups associated with DOC degradation, such as *Gemmataceae* and *Vicinamibacteraceae*, and nitrification, such as *Nitrospiraceae, Nitrosomonadaceae*, and *Nitrosopumilaceae,* were found throughout the sand bed. *Nitrosopumilaceae,* known to thrive under very low NH_4_^+^ concentrations, were found to be prevalent in the deeper depths where NH_4_^+^ was nearly depleted.^2^ These findings demonstrate the role of the entire sand bed in supporting key microbial communities and removal processes.^14,15^

In a recent study, we observed a significant release of DOC in the deeper layers of mature full-scale SSFs operating under low influent load of organic and inorganic matter, and microorganisms.^2^ A similar release has been reported in rapid sand filters and infiltration systems treating diverse source waters,^16,17^ suggesting that DOC release is a widely occurring phenomenon in biological filters. While the exact mechanism remains unclear, the release has been attributed to particulate organic carbon (POC) degradation, cell lysis, or solubilization. In these observations, released carbon was subsequently removed in the deeper depths. In a stable isotope labeling experiment using glucose as a proxy for easily biodegradable carbon, glucose removal was dominated by bacterial uptake over mineralization, with a substantial part likely retained as carbon reserves.^13^ These findings suggest that interactions between physical-chemical and biological processes in deeper filter layers may create temporary carbon sinks that later release stored carbon, with implications for long-term filter stability and design.

This study aimed to investigate the occurrence and potential pathways of DOC release in SSFs and whether this release depends on the filter age. To address this, mature full-scale SSFs operating at Dutch drinking water utilities and young laboratory-scale filters were studied. The full-scale filters operate as the final treatment step and thus receive water with low organic and inorganic matter and a low microbial load. To overcome the challenges of studying biochemical processes under these low-loading conditions, the laboratory filters were operated with a higher load of biodegradable DOC, NH_4_^+^, and PO_4_^3–^. Chemical parameters were monitored at different depths of full-scale and laboratory filters. The liquid chromatography organic carbon detection (LC-OCD) method was employed to characterize the organic carbon fractions contributing to DOC changes.^18,19^

## 2 Materials and Methods

### 2.1 Description and Sampling of Full-Scale SSFs

The drinking water treatment plant in Scheveningen (The Netherlands) of drinking water company Dunea receives raw water from the river Meuse and is further treated by managed aquifer recharge in the dunes, pellet softening, aeration, and rapid sand filtration with powdered activated carbon dosing. Further, the water flows through SSFs operated as a final polishing step before distribution to remove microbial growth-promoting compounds such as DOC and NH_4_^+^. Two mature full-scale SSFs from the same production line were examined in this study and have been producing drinking water for the last 28 years without sand replacement. Here on, the two full-scale filters will be termed F-SSF1 and F-SSF2. The sand bed heights of F-SSF1 and F-SSF2 are 95 and 85 cm, respectively, with an effective sand grain size of 0.3–0.6 mm. The filters consisted of supernatant height of 80–100 cm and operated at an average hydraulic loading rate of 0.4 m^3^/m^2^/h. The F-SSFs are operated indoors and the in situ water temperature at the treatment plant is mostly stable between 10 and 12 °C, as seasonal fluctuations are regulated during dune filtration. Specific operational and design parameters for the filters are listed in Table S1.

The water used to measure chemical parameters was sampled from both filters at influent, effluent, and five different depths of 5, 20, 30, 45, and 65 cm (measured from the top of the sand bed). The water was collected weekly over a 6-month period using sampling ports provided on the filter wall with 30 cm-long pipes penetrating the sand bed. The samples were collected once a week over a period of 5 months.

### 2.2 Operation and Sampling of Laboratory SSFs

Duplicate laboratory columns with a diameter of 4 cm consisted of a sand bed height of 85 cm, with an additional 5 cm under-drainage (5–7 mm gravel) to allow free passage of filtered water. The two laboratory columns, referred to as L-SSF1 and L-SSF2, were filled with freshly washed sand of different grain sizes: 0.45 mm (uniformity coefficient (UC) = 1.63) and 0.9 mm (UC = 1.51), respectively. L-SSF1 represented the grain size typically used in full-scale SSFs, while L-SSF2 with a coarser grain size was designed to represent groundwater filters. L-SSFs were operated at a controlled temperature of 19–21 °C for a period of 6 months at an average hydraulic loading rate of 0.5 m^3^/m^2^/h.

Nonchlorinated tap water from a multistage treatment scheme at a Dutch drinking water treatment plant was used as the influent for the laboratory filters. The DOC concentration in the tap water ranged from 1.8 to 2.5 mg/L, mainly comprising of neutrals, building blocks, biopolymers, and humics (Table S2). This residual DOC was not degraded during multistage treatment, indicating its recalcitrant nature. The concentrations of NH_4_^+^ and PO_4_^3–^ were below 0.01 mg/L. The tap water was supplemented with easily biodegradable carbon as a mixture of carboxylic acids, sodium acetate (NaC_2_H_3_O_2_), sodium oxalate (Na_2_C_2_O_4_), and sodium formate (NaCHO_2_) (1:1:1) (Merck chemicals), contributing to 1.5 mg/L of DOC, resulting in a final DOC concentration of 3.3–4 mg/L. NH_4_^+^ was added as ammonium chloride (NH_4_Cl) (Merck chemicals) to a concentration of 1 mg/L, and PO_4_^3–^ as potassium dihydrogen phosphate (KH_2_PO_4_^–^) (Merck chemicals) to reach 0.015 mg/L PO_4_^3–^–P according to the C:N:P molecular ratio of bacteria and biomass (100:10:1) (Table S3).

A supernatant water layer of 80 cm was maintained above the sand layer. The filters were covered with aluminum foil to exclude light and to avoid algal growth. The water samples were collected from influent, effluent, and five different depths of 5, 20, 35, 55, and 65 cm using sampling ports provided along the height of the columns. The samples were collected once a week over a period of 6 months.

### 2.3 Analytical Methods

#### 2.3.1 DOC

DOC was measured with a Shimadzu TOC-VCPH/CPN analyzer (limit of detection [LOD] = 0.1 mg/L) immediately or within 1 day after sampling. 30 mL of the sample was filtered through 0.45 μm filters (SPARTAN, Whatman, Germany) that had been flushed twice with demineralized water.

Liquid chromatography-organic carbon detection (LC-OCD; reporting limit-100 μgC/L) was used to assess the changes in the composition of DOC. The carbon fractions are distinguished based on their molecular weight (MW) from largest to smallest: biopolymers (proteins and polysaccharides), humic substances, building blocks, low-molecular-weight (LMW) acids, and neutrals. The analysis was conducted at Het Waterlaboratorium (Haarlem, The Netherlands) following a procedure described elsewhere.^45^

Historical influent data from F-SSFs showed AOC concentrations of <6 μg C/L (Table S1), well below the LC-OCD reporting limit of 100 μg C/L. Therefore, LC-OCD measurements were conducted on L-SSFs supplemented with higher AOC concentrations.

#### 2.3.2 NH_4_^+^, and PO_4_^3–^

The concentrations of NH_4_^+^ (LOD = 0.01 mg N/L), NO_2_^–^ (LOD = 0.01 mg N/L), NO_3_^–^ (LOD = 0.1 mg N/L), and PO_4_^3–^ (LOD = 0.001 mg/L) in the filtered water (<0.22 μm) samples were determined using Ion Chromatography (Dionex ICS-2100, Thermo, USA) equipped with an AS17-Column.

#### 2.3.3 pH and Dissolved Oxygen

Dissolved oxygen (DO) concentrations, pH, and temperature were measured directly in the water from sampling ports using a HQ40D portable multimeter (HACH), with a tube from each tap leading directly into a 500 mL polypropylene bottle that overflowed continuously.

#### 2.3.4 Analysis of Sand-Associated Biomass

Total adenosine triphosphate (tATP) was measured on filter sand with 1 g of wet media sample using Luminultra Deposit and Surface analysis test kit, following the manufacturer’s instructions. Measurements were taken with a luminometer.

### 2.4 Calculation of Carbon Released from Biomass

The heterotrophic and nitrifying biomass produced in laboratory SSFs were calculated from their respective biomass yield and substrates utilized as follows (eq 1):

Biomassproduced(X)=Biomassyield(Y)×Substrateutilized(S) 1

where yield (*Y*) was considered as 0.71 g volatile suspended solids (VSS)/g sodium acetate for heterotrophs^20,21^ and 0.2 g VSS/g NH_4_^+^–N for nitrifiers.^22^ Heterotrophs utilize both easily and slowly biodegradable DOC, depending on influent water characteristics. In this study, the influent of the laboratory SSFs was supplemented with easily biodegradable DOC. Therefore, the reduction in DOC was attributed to this fraction and considered as the substrate for heterotrophs. Meanwhile, the substrate for nitrifiers was NH_4_^+^–N consumed during nitrification. Based on the biomass concentration, the carbon content in biomass was calculated considering a biomass composition of CH_1.8_O_0.5_N_0.2_.

### 2.5 Statistical Analysis

Statistical analysis was performed on all quantitative data using one-way ANOVA, followed by the Bonferroni post hoc correction.

## 3 Results

### 3.1 DOC Release Observed in Mature Full-Scale SSFs

Full-scale SSFs operating as the final treatment step received an influent DOC concentration of 3.8–4.2 mg/L. A substantial DOC reduction of 0.6–0.8 mg of C/L occurred in the top 5 cm of the filter (Figure 1). However, between 20 and 60 cm depth, a significant increase in DOC (*p* < 0.05) was noted, with a release of around 0.36 mg C/L in both filters. The released DOC fraction was subsequently removed in the deeper layers. Hence, the release would have remained unnoticed if merely effluent DOC concentrations were monitored. This variation of DOC over depth was consistently observed throughout the 5-month sampling period.

**Figure 1:**
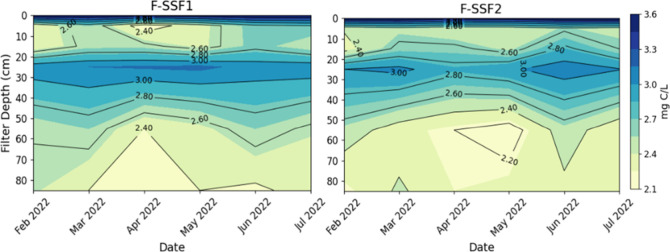
Depth profiles of DOC in mature full-scale SSFs: F-SSF1 and F-SSF2 between February and July 2022. Samples were collected weekly over a 6-month period (*n* = 26), and measurements were performed in triplicate.

These full-scale SSFs are located downstream of several treatment steps, including dune passage and rapid sand filtration. As a result, the influent water is low in organic and inorganic load, with AOC concentrations below 5 μg C/L, indicative of oligotrophic conditions.^2^ Such a low substrate availability poses challenges for studying carbon cycling in these systems.

Concentrations of NH_4_^+^, DO, and pH decreased with depth (Figure S1). Despite a low NH_4_^+^ concentration (<0.01 mg N/L) in the influent, a minor yet significant decrease (*p* < 0.05) was noted between 5 and 55 cm. Both DO and pH slightly decreased in the top 40 cm and then stabilized in the deeper depths.

### 3.2 Laboratory SSFs Mirror Full-Scale Findings

In this study, L-SSFs were operated with tap water supplemented with higher concentrations of easily biodegradable DOC than typically present in the influent of full-scale SSFs, to investigate the dynamics of DOC removal. During the initial operation phase, only a minor DOC decrease (<5%) was observed across the filter depth, as fresh, clean sand was used as the filter medium. From day 70 onward, a substantial decrease of DOC occurred primarily in the top 5 cm (0.63 mg/L), and this removal remained consistent over time. However, a pronounced DOC increase was observed at 55 cm depth, with average DOC concentrations 0.31 mg/L higher than at the 35 cm sampling point. The released DOC was subsequently removed in deeper layers between 65 and 95 cm.

After day 95, DOC depth profiles showed a consistent pattern, indicating stable removal across the filter depth. The increase in DOC removal capacity corresponded with increasing biomass concentrations (measured by tATP) at 5, 20, and 55 cm depth over time (Figure S2). Biomass distribution showed clear stratification with the highest concentrations in the top 5 cm. These observations suggest a key role of biological processes in DOC removal in combination with physical-chemical processes.

### 3.3 Nitrification and PO_4_^3–^ Removal in Laboratory Filters

A substantial DOC decrease occurred in the top 5 cm from day 70 onward, followed by release and subsequent reuptake in the deeper layers. The DOC depth profiles stabilized from day 95. While NH_4_^+^ removal began below 5 cm depth and reached complete removal (0.98 mg/L) in the first 45 cm depth after 103 days of operation (Figures S3 and S4). The depth profiles of DOC and NH_4_^+^ exhibited stable patterns after days 95 and 103, respectively, in L-SSFs (Figures 2 and S4), indicating the establishment of steady-state conditions. Accordingly, the depth profiles of DOC, NH_4_^+^, PO_4_^3–^, and pH measured between days 120 and 123 are presented in Figure 3 as representative of the steady state in this study.

**Figure 2:**
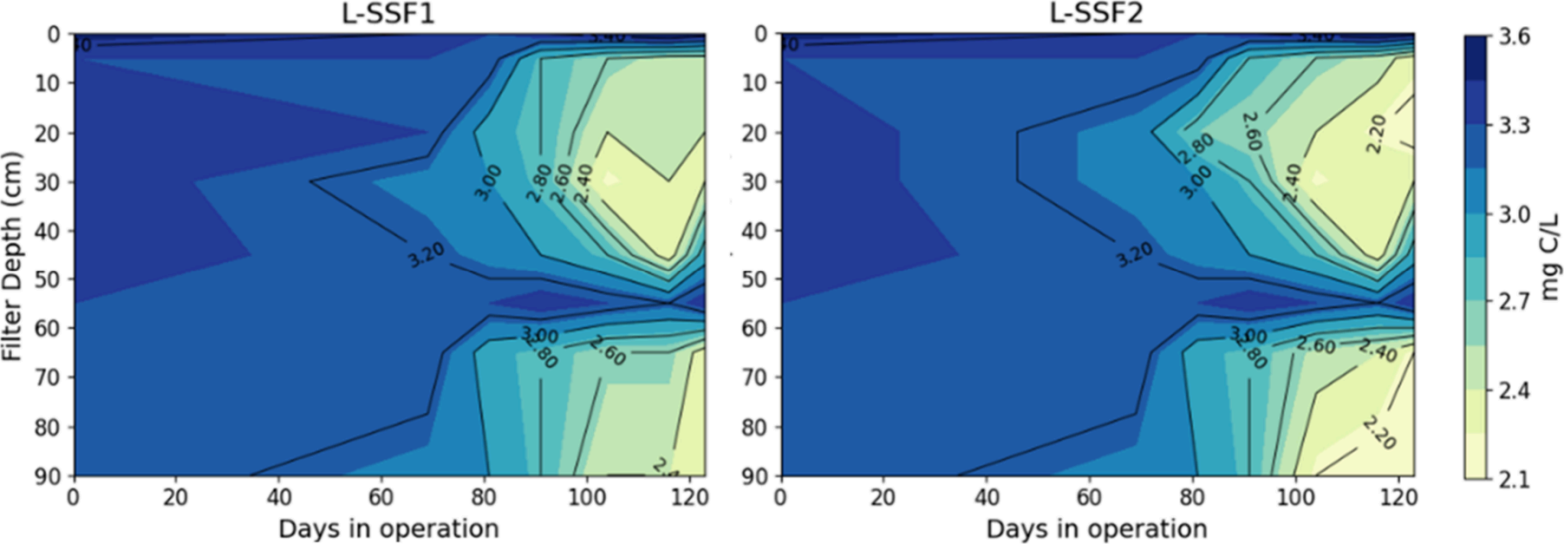
Depth Profiles of DOC in laboratory SSFs: L-SSF1 and L-SSF2 over time of operation. Samples were collected weekly over a 6-month period (*n* = 26), and measurements were performed in triplicate.

**Figure 3:**
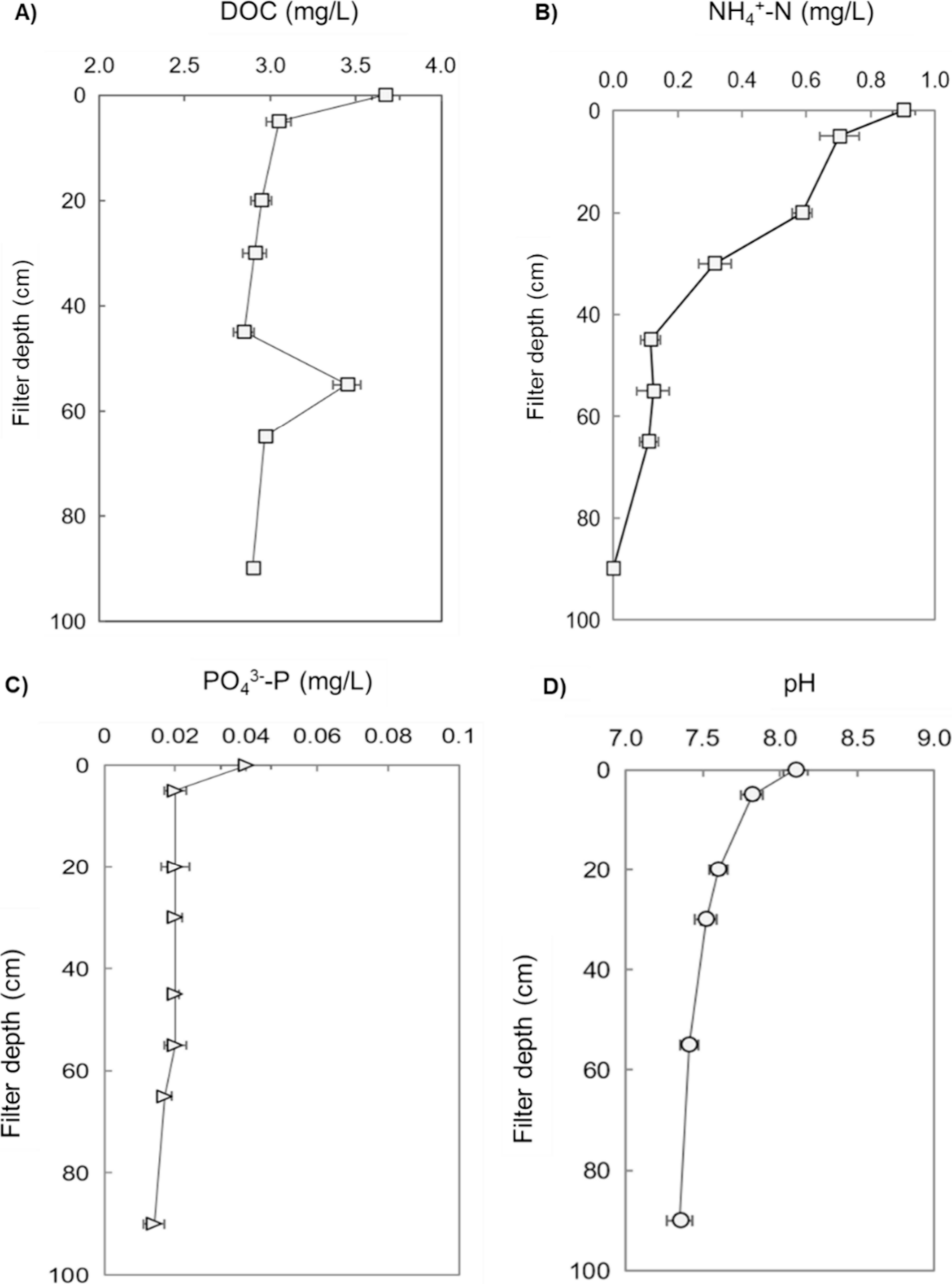
Depth profiles of dissolved organic carbon (DOC, A), ammonium (NH_4_^+^, B), phosphate (PO_4_^3–^, C), and pH (D) in laboratory SSFs between days 120 and 123 of operation under steady-state conditions (*n* = 4). Data represent mean values, and error bars indicate the standard deviation. Each measurement was conducted in triplicate.

NH_4_^+^ removal was accompanied by an increase in the NO_2_^–^ and NO_3_^–^ concentrations, along with a decrease in pH and DO (Figures 3B and S3), collectively indicating nitrification. It is noteworthy that 0.21 mg/L NH_4_^+^ was not recovered as NO_2_^–^ or NO_3_^–^ in the effluent; i.e., this fraction could not be linked to nitrification. This did, however, correspond to the observed DO consumption, which is too low for complete nitrification of incoming NH_4_^+^. PO_4_^3–^ concentration in the influent was already low (0.04 mg/L), yet a significant decrease (*p* < 0.05) of 0.02 and 0.01 mg/L was noted in the top 5 cm and between 55 and 90 cm depths, respectively (Figure 3C).

### 3.4 Depth-Specific Composition of DOC

LC-OCD analysis was used to characterize the contribution of different organic carbon fractions to DOC changes in laboratory SSFs. DOC removal across the filter depth stabilized after day 95, indicating steady-state conditions. Therefore, water samples from different depths were analyzed by LC-OCD on day 106 of operation (Figure 4). The influent DOC consisted of LMW acids (0.24 mg/L), LMW neutrals (0.35 mg/L), building blocks (1.0 mg/L), biopolymers (0.16 mg/L), and humic substances (0.92 mg/L). The concentrations of LMW acids and building blocks in the influent were higher than those in the background tap water, reflecting the addition of easily biodegradable carbon.

**Figure 4:**
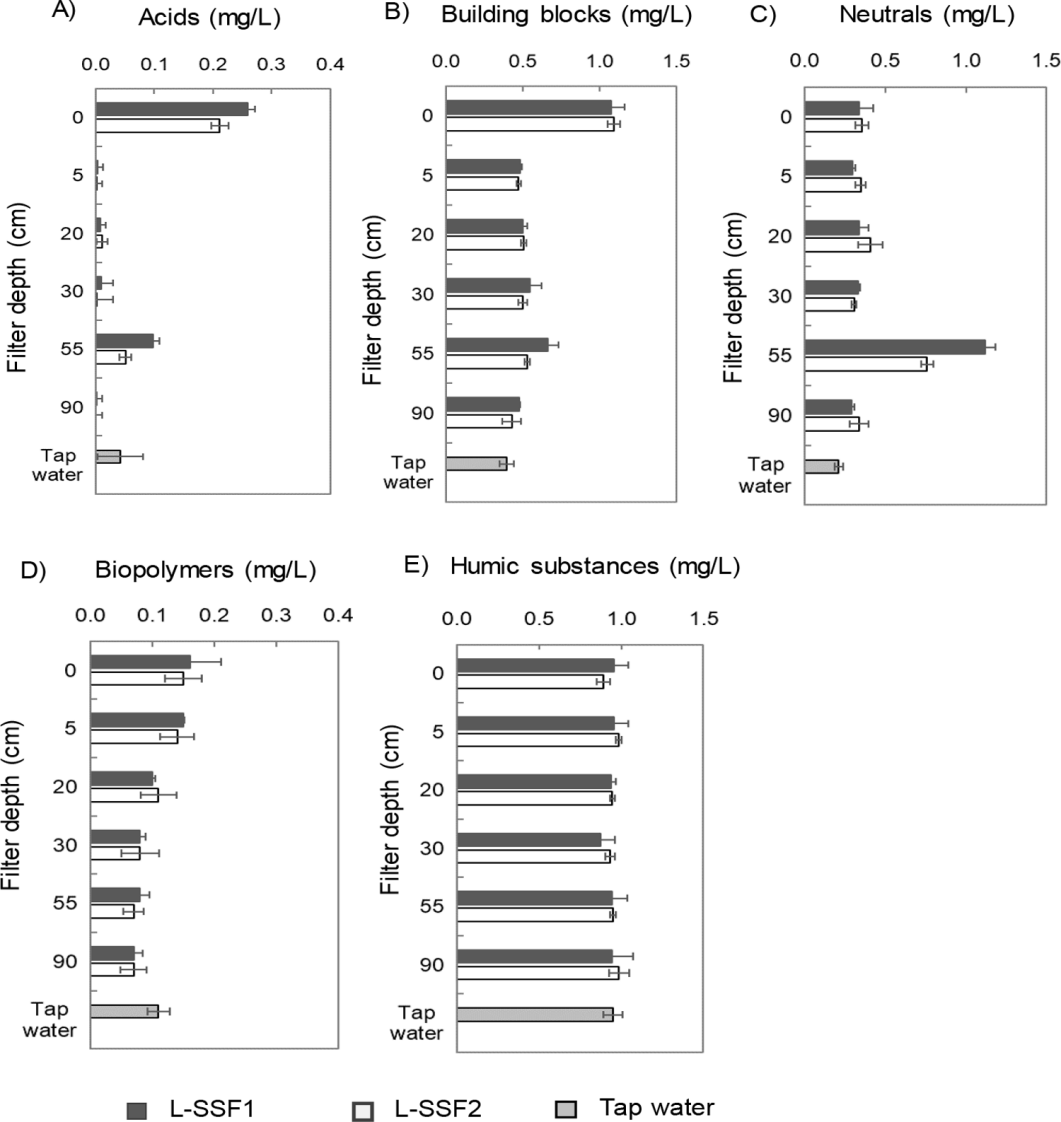
Concentrations of (A) acids, (B) building blocks, (C) neutrals, (D) biopolymers, and (E) humic substances in tap water and the over depth of laboratory SSFs after 106 days of operation.

DOC removal in the top 5 cm was due to a decrease in the level of acids (0.23 mg/L) and building blocks (0.6 mg/L). Biopolymers concentration decreased linearly between 20 and 90 cm, up to a total removal of 0.1 mg/L in the effluent. The released DOC concentration (0.83 mg/L) at 55 cm depth was caused by an increase in acids (0.1 mg/L) and neutrals (0.81 mg/L) (Figure S5). It is important to note that the LC-OCD reporting limit is 100 μg C/L (0.1 mg/L), meaning that acid concentrations between 5 and 90 cm are at or below this threshold and should be interpreted with analytical uncertainty. The concentration of neutrals at 55 cm exceeded that of the influent, suggesting that processes within the sand bed contributed to the increase. The released acids and neutrals were subsequently removed between 55 and 90 cm. Notably, the (background) concentrations of humic substances and neutrals in the influent stayed consistent throughout the filter depth, implying that these compounds were not removed by either physical-chemical or biological processes.

## 4 Discussion

### 4.1 Release of Low-Molecular-Weight Organic Carbon in SSFs

In mature full-scale filters, DOC release was consistently observed at a depth of 20–60 cm. Also, after a start-up period of two months, the young laboratory SSFs started demonstrating a stable release of DOC at 55 cm. These observations in both young and mature SSFs suggest that DOC release in SSFs is independent of biofilm maturity. Figure 5 shows a schematic illustration of the proposed processes occurring at different filter depths. The top 5 cm, in SSF literature commonly referred to as the *Schmutzdecke*, was found to consistently remove easily biodegradable fractions such as acids and building blocks. The stoichiometric calculations showed that the DO decrease in this layer was similar to the estimated oxygen needed to degrade the observed change in DOC (Table S4). Thus, DOC removal is attributed to the activity of heterotrophs that utilize these compounds for assimilation and respiration.^23,25^ Although neutrals could serve as a substrate for microbial growth,^26^ the steady levels between 0 and 40 cm indicate their recalcitrant characteristics. The nitrogen balance (Table S5) at 5 cm indicated that only a fraction of NH_4_^+^ (0.05 mgN/L) was converted to NO_2_^–^ and NO_3_^–^, indicating that the remaining NH_4_^+^ (0.21 mgN/L) may be assimilated by the fast-growing heterotrophs.^24^ The observed PO_4_^3–^ removal in this layer may also be attributed to the growth of new biomass. This is supported by the elemental molar ratio of removed DOC, NH_4_^+^, and PO_4_^3–^, which closely matches the microbial growth ratio of 100:10:1.^27^

**Figure 5:**
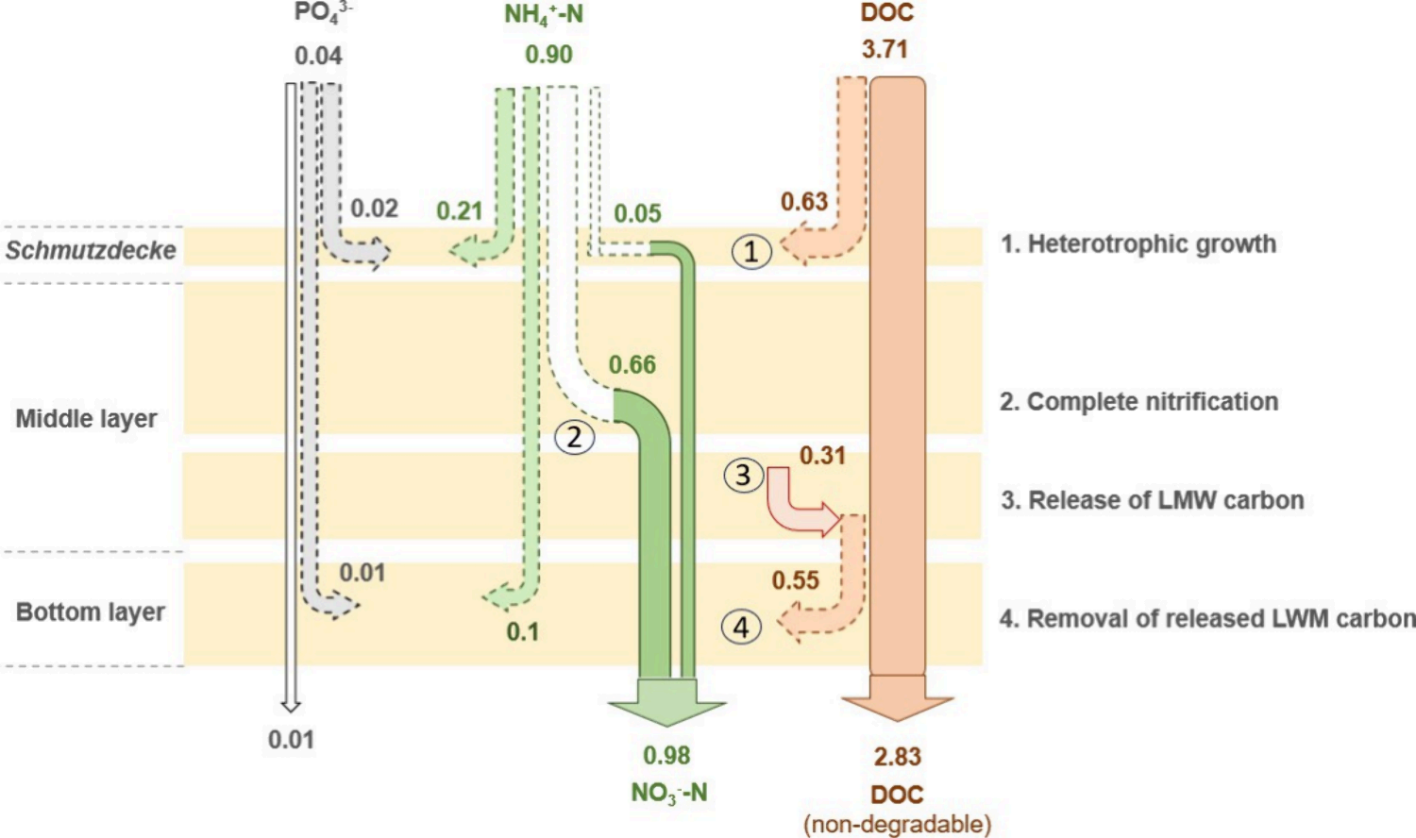
Schematic illustration of the fate of DOC, NH_4_^+^, and PO_4_^3–^ (mg/L) at different filter depths between days 120–123 of operation. Colored dotted arrows indicate the consumption of NH4^+^ and PO4^3–^ for heterotrophic growth, while white dotted arrows indicate the consumption of NH4^+^ by nitrification.

Nitrification primarily occurred beyond the *Schmutzdecke*, at a depth lower than that of DOC removal, leading to a deeper infiltration of NH_4_^+^ and NO_2_^–^. The spatial separation between DOC and NH_4_^+^ removal is probably due to the higher growth rate of heterotrophs compared to nitrifiers, along with the availability of high concentrations of easily biodegradable carbon in the top layers that can be utilized by the heterotrophs.^28^ Moreover, heterotrophs preferentially use NH_4_^+^ as a nitrogen source over NO_2_^–^ and NO_3_^–^, which may have reduced NH_4_^+^ availability for nitrifiers,^29^ slowing nitrification in the top layers. Nitrifiers such as *Nitrospiraceae* and *Nitrosomonadaceae* were found to be abundant in both top and deeper layers of SSFs.^2,30^ Nitrifying bacteria have been found to coexist with heterotrophic bacteria, which benefit from the carbon produced by nitrifiers,^31^ while the nitrifiers benefit from the production of biofilm and consumption of inhibitory metabolites by heterotrophs.^32^

The LMW acids and neutrals released at 55 cm were completely removed in the subsequent sand layers, together with a significant removal of a fraction of PO_4_^3–^ (*p* < 0.05). Neutrals did not decrease below the nonbiodegradable concentration present in tap water, and humic substances remained unchanged throughout the filter depth. This suggests that both physical-chemical and biological processes in SSFs were unsuccessful in removing the complex and recalcitrant neutrals and humic substances present in tap water. The released easily biodegradable acids and neutrals may have been mainly removed biologically in the bottom layers, although physical-chemical processes may also have contributed.^7,26,33^ It is worth noting that with sufficient filter depth, DOC cycling does not compromise effluent quality. In fact, it can reduce PO_4_^3–^ concentration to ultralow levels. Additionally, the released carbon could enhance heterotrophic activity, potentially aiding the co-removal of other contaminants from drinking water. Heterotrophs and nitrifiers have been identified as degraders of organic micropollutants (OMPs)^34,35^ and could benefit from an additional carbon source. For instance, in rapid sand filters, increased loading of dissolved organic matter enhanced biodegradation of paracetamol by providing supplementary carbon for heterotrophic degraders.^36^

### 4.2 Potential Pathways of DOC Release

The release of easily biodegradable DOC in the deeper layers of SSFs observed in this study may be explained by several mechanisms. One explanation could be that DOC originates from decaying bacterial cells. As water flows through the filter, cells may detach from biofilms in the top layers and become trapped in deeper layers of the filters.^2,37^ High concentrations of dead cells were reported in water sampled from deeper depths of mature full-scale SSFs.^2^ This biomass could release labile carbon as a byproduct of cellular metabolism or through cell decay,^17^ which may then be consumed by microorganisms in the bottom layers of the filter, stimulating microbial growth.^16,17^

Interestingly, the release of DOC coincides with depths where nitrification is nearly complete in both mature and young filters. Ammonia-oxidizing bacteria (AOB), a key group of nitrifiers, have been found to release DOC during growth in carbon-limited marine ecosystems.^32,38,39^ Thus, the release could be a characteristic of metabolically active nitrifiers and not an artifact of experimental conditions. A release of amino acids by exponentially growing *Nitrosopumilus* cells was observed, suggesting that DOC release is a typical behavior in the nitrifier population.^34^ This labile carbon can serve as an additional carbon source for other microbes, supporting microbial loops in natural environments.

Another possibility is that nitrifiers from middle layers are transported downward to depths where the NH_4_^+^ and NO_2_^–^ are limited. The prevalence of nitrification in middle layers is indicated by an increase in NO_3_^–^ and a decrease in pH at these depths. Nitrifiers such as *Nitrospiraceae, Nitrosomonadaceae*, and *Nitrosopumilaceae* have been shown to be more abundant in sand from deeper depths compared to top layers in full-scale SSFs.^2^ However, it is crucial to note that 16S rRNA gene sequencing, which is used to identify and quantify these communities, cannot distinguish between live and dead cells and may still detect DNA from non-viable cells. As cells are transported to deeper layers, they may starve and die due to NH_4_^+^ and NO_2_^–^ limitation, with their necromass serving as a source of labile carbon for heterotrophic bacteria. This phenomenon, where *Nitrospira* necromass supports heterotrophic growth has been documented in drinking water distribution networks.^40^

In this study, NH_4_^+^ concentrations in the deeper depths of full-scale filters were found to be below 0.005 mg/L, and around 0.1 mg/L in laboratory filters, suggesting that the NH_4_^+^ limitation might not be as critical in the latter. The estimated DOC release from decaying nitrifying biomass was 0.11 mg C/L in laboratory filters. Even if all nitrifiers decayed, a highly improbable scenario, this would only account for a fraction of the observed 0.31 mg C/L DOC release. It is also important to consider the variability in carbon content across nitrifier communities. A higher carbon content has been reported for nitrite-oxidizing bacteria (NOB) compared to ammonia-oxidizing archaea (AOA),^38,41,42^ and carbon content also varies across different growth phases.^35^ When considering the decay of heterotrophic cells, the estimated release of organic carbon from their biomass is higher, around 0.34 mg C/L. While theoretically possible, it is unlikely that heterotrophic biomass decays uniformly at a depth of 55 cm, as the released cells would have to be transported from the *Schmutzdecke,* where heterotrophic populations dominate.

An alternative hypothesis is the conversion of slowly biodegradable carbon fractions, such as biopolymers, into easily biodegradable compounds, such as acids and neutrals. Biopolymers have been shown to serve as microbial substrates in oligotrophic conditions.^43,44^ Lautenschlager et al. suggested that polysaccharides may be removed biologically in deeper layers of SSFs due to the prolonged contact time between substrates and biofilms.^26^ However, the observed removal of biopolymers, around 0.1 mg/L, does not align with the sharp DOC release peak, making it an unlikely source for the observed DOC peak.

### 4.3 Implications for Practice and Future Research

This study provides the first evidence of the release of easily biodegradable carbon in the deeper layers of SSFs, observed in both mature full-scale and young laboratory filters. Together with previous reports of carbon release in infiltration systems and rapid sand filters, these findings highlight this as a widespread phenomenon of practical relevance.

Investigating DOC composition in full-scale filters was challenging due to the low load of easily biodegradable carbon. Nevertheless, DOC removal, release, and reuptake along the filter depth, as well as stratified NH_4_^+^ removal, were clearly observed. The lab-scale filters showed a similar trend during steady-state operation after 120 days at higher DOC and NH_4_^+^ levels, suggesting that these processes may occur in both systems despite differences in loading and biofilm age. These observations indicate that the potential pathways identified in laboratory filters may also be relevant for full-scale SSFs. However, full-scale SSFs operating for several years have established biofilms with different biomass concentrations, microbial communities, and extracellular polymeric substances (EPS) composition compared with laboratory filters and may influence carbon cycling processes. While established removal processes in mature SSFs can compensate for disturbances in filter depths or influent quality,^2^ potential long-term impacts of accumulated bacterial biomass and necromass on biological stability indicators such as microbial growth potential and biofouling potential require further investigation. Overall, these findings emphasize the importance of deeper layers for the filter performance and stability.

In this study, both full- and lab-scale filters demonstrated that DOC removal and complete nitrification occurred within the top 45 cm. From a biological stability perspective, SSFs could be designed with a shallower bed height. However, a full sand bed height of 1 m may be necessary for effective disinfection, particularly in young filters and after scraping. Before implementing specific design or operational modifications, it is essential to clarify the mechanisms underlying the carbon release in biological filters through long-term experiments. In particular, SSFs operated outdoors may experience greater variability in influent water quality and seasonal fluctuations, which can significantly influence carbon cycling processes. Stable isotopes provide a valuable tool for tracing dissolved organic matter in water treatment.^46^ Approaches include analyzing natural isotope ratios or using stable isotope labeling, where isotopes such as ^13^C are deliberately introduced as tracers.^47^ For example, ^13^C-labeled glucose has been used to investigate carbon transformations within SSFs.^13^ A comprehensive understanding of the biochemical processes and microbial taxa driving carbon cycling in filter beds is essential to improving SSF operation.

## 5 Conclusions

This study revealed that although the top *Schmutzdecke* layer effectively reduced the easily biodegradable fraction of DOC, such as acids and building blocks, the deeper filter layers released DOC as LMW acids and neutrals. This DOC release occurred at depths where nitrification was nearly complete and was observed in both mature full-scale and young laboratory-scale SSFs, suggesting that it is not solely dependent on the filter age. Potential pathways for the observed DOC release include release by bacterial cells trapped in deeper layers due to carbon or nitrogen limitation or conversion of slowly degradable carbon into easily biodegradable forms. Importantly, the released DOC was subsequently removed in the deepest layers, indicating an internal cycling of carbon that would go undetected if only effluent concentrations were monitored. This study presents the first evidence of biodegradable DOC release in the deeper layers of SSFs, urging further research to understand biochemical processes and carbon cycling throughout the filter bed and its implications for long-term filter stability and performance.

## Supplementary Material



## Notes

Supplementary figures and tables, and calculated mass balances of chemical parameters measured in filters (PDF)
