# Supplementary material for: Easily Biodegradable Organic Carbon Release in the Deep Bed of Slow Sand Filters
Source: ACS ES T Water. 2025 Oct 15;5(11):6961–9. doi: 10.1021/acsestwater.5c00932 (PMC12624717; doi:10.1021/acsestwater.5c00932)
Supplement: Supplementary file 1 [file ew5c00932_si_003.pdf]

Supplementary information

Easily biodegradable organic carbon release in deep bed of slow sand filters

Shreya Ajith Trikannad, [a,b] *Jan Peter van der Hoek,*[a,c] *Yuwei Huang,*[a] *Doris van Halem*[*,a]

[a]Department of Water Management, Delft University of Technology, Building 23, Stevinweg 1, 2628 Delft, The Netherlands

[b]Eawag, Swiss Federal Institute of Aquatic Science and Technology, 8600 Dübendorf, Switzerland

[c]Waternet, Korte Ouderkerkerdijk 7, 1096 AC, Amsterdam, the Netherlands

* Corresponding author.

E-mail address: D.vanhalem@tudelft.nl (D. van Halem)

1. Tables

Table S1 Operational parameters, influent and effluent characteristics of full-scale SSFs. Historical data refer to average and standard deviation of the biweekly measured concentrations between June 2020 and August 2022.

	Unit		Scraped filter	Control filter
Filter bed height	m		0.95	0.8
Height of supernatant	m		1	1
Filter area	m^2		2383	2359
Filtration rate	m/h		0.4	0.4
Grain size	mm		0.3-0.6	0.3-0.6
Age of media	years		28	28
Time since last scraping	years		5.3	4.6
Historical data		Influent	Effluent	
Temperature	ºC	14.2 ± 2.68	14.5 ± 2.68	14.3 ± 2.68
ATP	pg/ml	6.24 ± 0.08	1.5 ± 0.08	1.41± 0.07
ICC	cells/ml	2.66 x 105 ± 0.03	1.66 x 10^5 ± 0.03	1.70 x 10^5 ± 0.03
TCC	cells/ml	4.70 x 105 ± 0.04	2.70 x 10^5 ± 0.04	2.59 x 10^5 ± 0.03
AOC	µg C/L	5.45 ± 0.04	3.7 ± 0.04	3.8 ± 0.05
DOC	mg C/L	3.65 ± 0.02	2.65 ± 0.02	2.7 ± 0.03
NO_3^-	mg N/L	0.98 ± 0.01	1.20 ± 0.01	1.25 ± 0.02
NH_4^+	mg N/L	0.009	0.006	0.006
NO_2^-	mg N/L	0.001	0.001	0.001
Turbidity	NTU	2.11 ± 0.01	1.36 ± 0.01	1.39 ± 0.01
Coliforms	CFU/L	0 ± 2	0	0
Escherichia coli	CFU/L	0 ± 1	0	0
Sulfite-reducing clostridia	CFU/L	0 ± 1	0	0

CFU- Colony Forming Unit

Table S2 Dissolved organic carbon (DOC) composition in tap water determined by Liquid chromatography – organic carbon detection (LC-OCD).

DOC composition in tap water	
**Fractions**	**Concentration (mg/L)**
Low molecular-weight acids	0.042 ± 0.039
Low molecular-weight neutrals	0.210 ± 0.028
Building blocks	0.397 ± 0.046
Biopolymers	0.110 ± 0.018
Humics	0.951 ± 0.057

Table S3 Composition and concentration of compounds dosed in tap water.

Parameter	Dosed concentration (mg/L)	Compound	Chemical concentration (mg/L)
DOC	0.85	Sodium acetate ($NaC_2H_3O_2$)	1.607
		Sodium formate ($NaCHO_2$)	1.606
		Sodium oxalate ($Na_2C_2O_4$)	1.582
NH_4^+-N	1	Ammonium chloride (NH_4Cl)	3.821
PO_4^{3-}-P	0.015	Pottasium dihydrogen phosphate (KH_2PO_4)	0.048

Table S4 Measured and calculated dissolved oxygen consumption for DOC and NH_4^+ removal at different depths of laboratory SSFs.

	Depths	Δ DO (mg/L)	Δ DOC (mg/L)	Δ NH_4^+ (mg/L)	Δ DO_{DOC} (mg/L)	Δ DO_{NH4+} (mg/L)
Laboratory SSFs	Top (0-5 cm)	-0.45	-0.63	-0.21	-0.35	-0.16
	Middle (5-55 cm)	-0.50	0.31	-0.66	-0.05	-1.50
	Bottom (55-90 cm)	-0.29	-0.55	-0.08	-0.34	-0.13

Δ DO: determined DO removal

Δ DOC: determined DOC removal

Δ NH_4^+: determined NH_4^+ removal

Δ DO_{DOC}: calculated DO consumption for DOC removal, at 1.07 mg DO/1 mg DOC

Δ DO_{NH4+}: calculated DO consumption for NH_4^+ removal, at 4.57 mg DO/mg NH_4^+-N

Table S5 Mass balances of NH^{+}, NO_2^{-} and NO_3^{-} at different depths calculated using data from the last four days of the experiment in laboratory SSFs.

	Depths	Δ NH_4^+ (mg/L)	Δ NO_2^- (mg/L)	Δ NO_3^- (mg/L)	ΔN
Laboratory SSFs	Top (5 cm)	-0.26	0.01	0.04	-0.21
	Middle (55 cm)	-0.66	0.06	0.64	0.04
	Bottom (90 cm)	-0.08	0.01	0.22	0.16

2. Figures

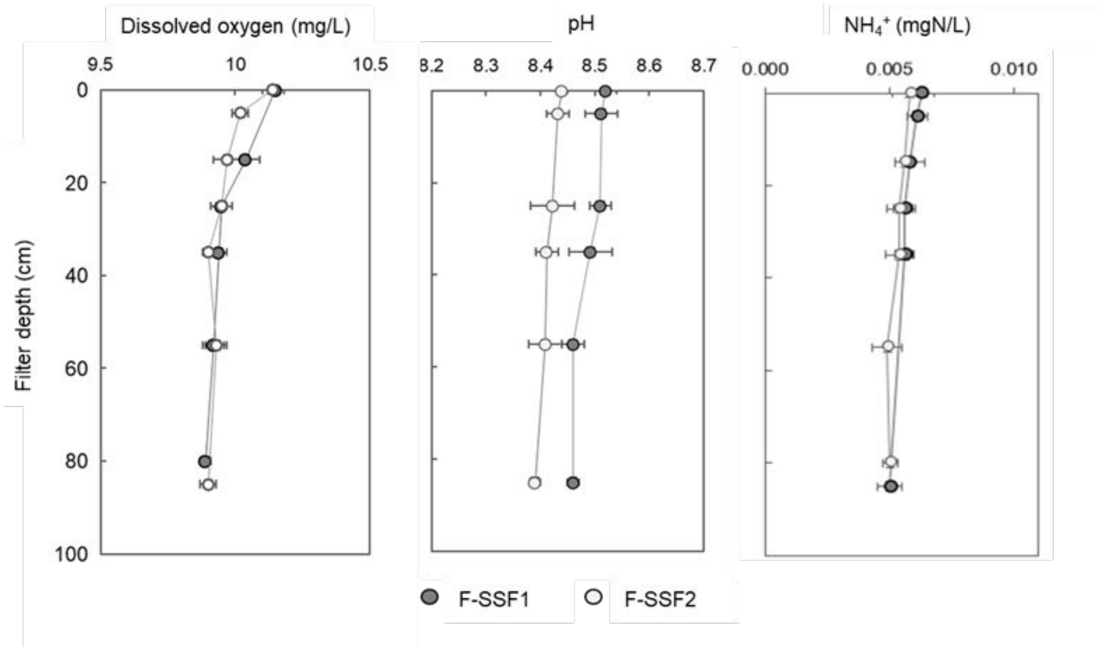

Figure S1 Depth profiles of (A) dissolved oxygen, (B) pH and (C) NH4+ in full-scale SSFs. Measurements were carried out in triplicates.

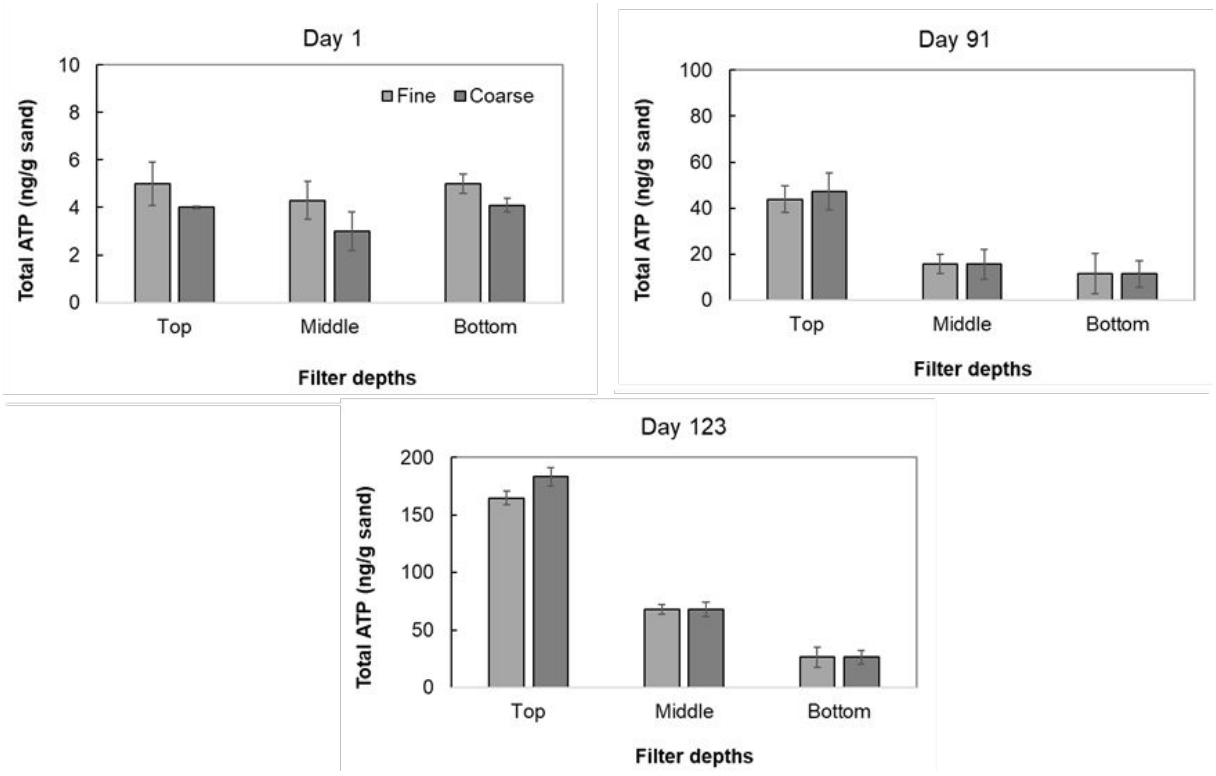

Figure S2 Total biomass on sand determined by total ATP from top, middle and bottom layers of laboratory SSFs over time of operation.

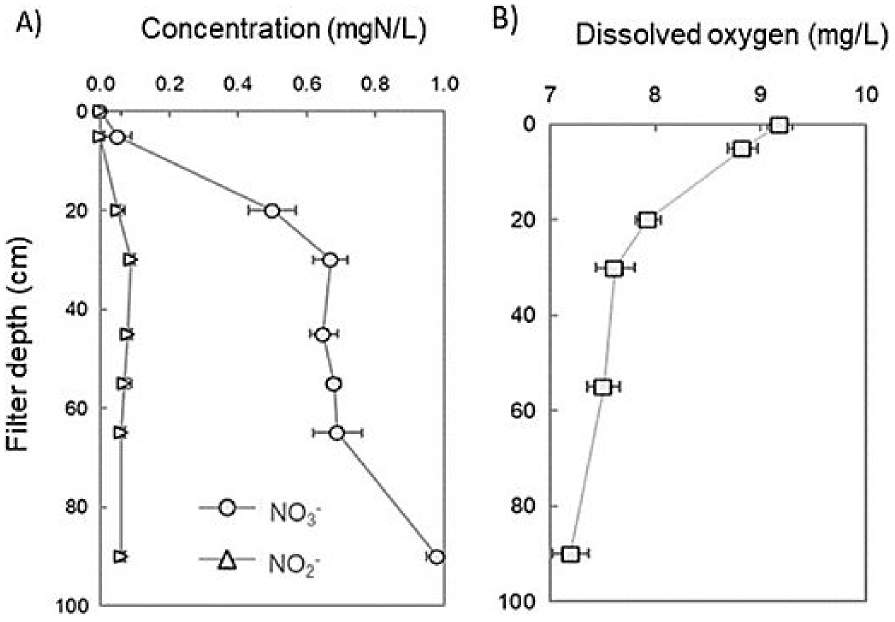

Figure S3 Depth profiles of (A)NO_3^- and NO_3^- and (B) dissolved oxygen on the last four days of the experiment in laboratory SSFs. Measurements were carried out in triplicates.

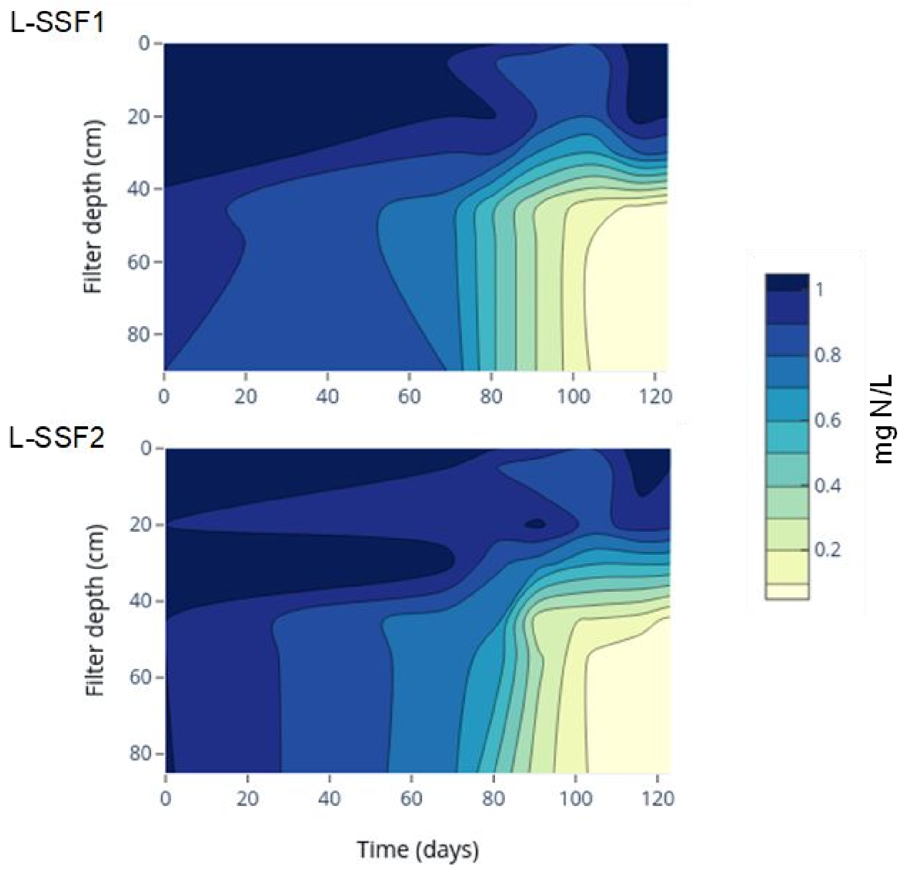

Figure S4 Depth profiles of NH_4^+ in laboratory SSFs: L-SSF1 and L-SSF2 over time of operation. Samples were collected weekly over a 6-month period (n = 26), and measurements were performed in triplicate

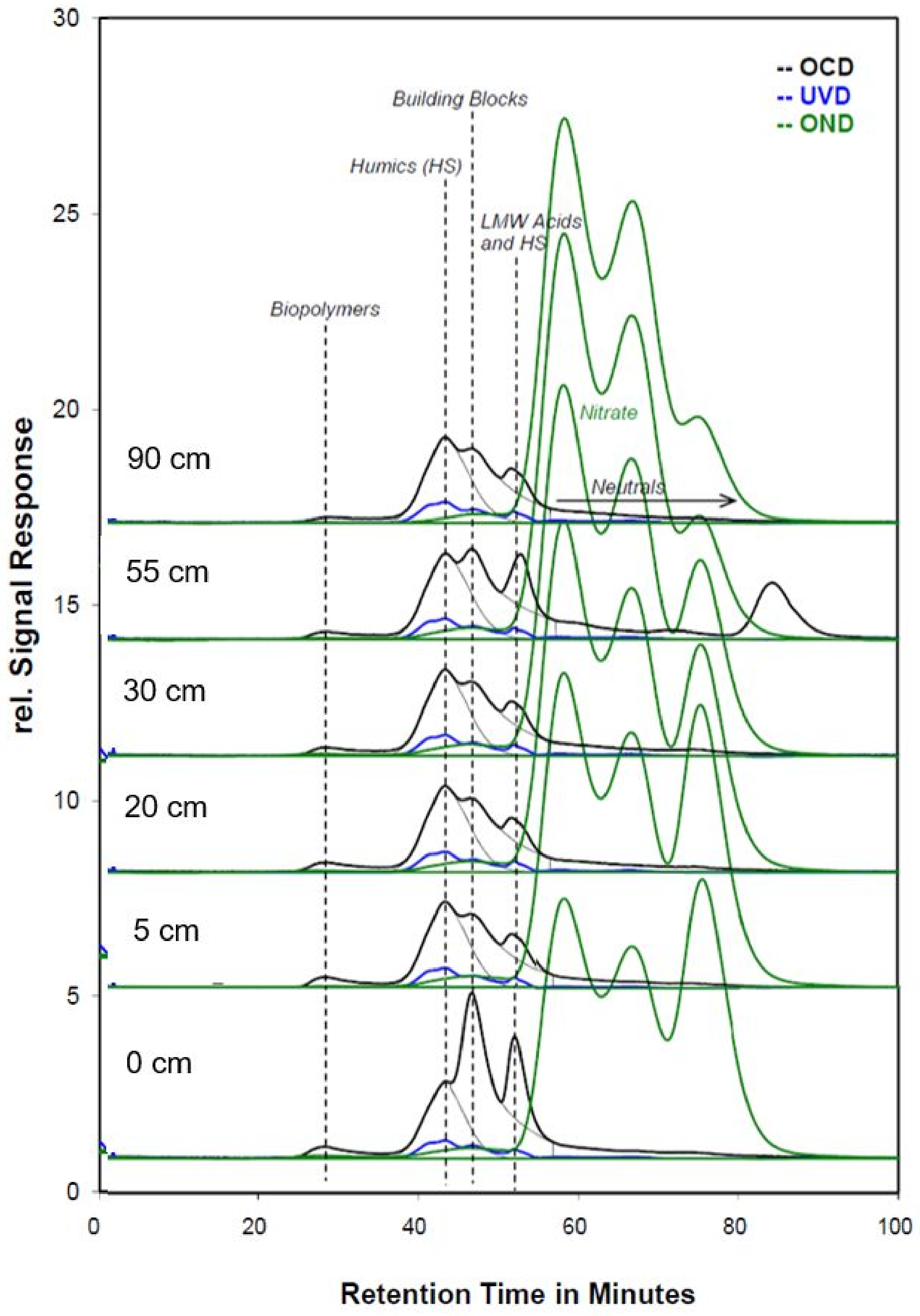

Figure S5 LC-OCD chromatograms illustrating DOC fractions in water samples collected at different depths of laboratory SSFs.
